# Fructose Intake, Hypertension and Cardiometabolic Risk Factors in Children and Adolescents: From Pathophysiology to Clinical Aspects. A Narrative Review

**DOI:** 10.3389/fmed.2022.792949

**Published:** 2022-04-12

**Authors:** Marco Giussani, Giulia Lieti, Antonina Orlando, Gianfranco Parati, Simonetta Genovesi

**Affiliations:** ^1^Cardiologic Unit, Istituto Auxologico Italiano, Istituto Ricovero Cura Carattere Scientifico (IRCCS), Milan, Italy; ^2^School of Medicine and Surgery, University of Milano-Bicocca, Milan, Italy

**Keywords:** children, fructose, hypertension, cardio-metabolic risk factors, obesity, sugar-sweetened beverages

## Abstract

Arterial hypertension, dyslipidemia, alterations in glucose metabolism and fatty liver, either alone or in association, are frequently observed in obese children and may seriously jeopardize their health. For obesity to develop, an excessive intake of energy-bearing macronutrients is required; however, ample evidence suggests that fructose may promote the development of obesity and/or metabolic alterations, independently of its energy intake. Fructose consumption is particularly high among children, because they do not have the perception, and more importantly, neither do their parents, that high fructose intake is potentially dangerous. In fact, while this sugar is erroneously viewed favorably as a natural nutrient, its excessive intake can actually cause adverse cardio-metabolic alterations. Fructose induces the release of pro-inflammatory cytokines, and reduces the production of anti-atherosclerotic cytokines, such as adiponectin. Furthermore, by interacting with hunger and satiety control systems, particularly by inducing leptin resistance, it leads to increased caloric intake. Fructose, directly or through its metabolites, promotes the development of obesity, arterial hypertension, dyslipidemia, glucose intolerance and fatty liver. This review aims to highlight the mechanisms by which the early and excessive consumption of fructose may contribute to the development of a variety of cardiometabolic risk factors in children, thus representing a potential danger to their health. It will also describe the main clinical trials performed in children and adolescents that have evaluated the clinical effects of excessive intake of fructose-containing drinks and food, with particular attention to the effects on blood pressure. Finally, we will discuss the effectiveness of measures that can be taken to reduce the intake of this sugar.

## Introduction

The younger generations are increasingly subjected to early and significant exposure to sugar, and fructose in particular (1). Fructose is associated with the development of obesity (2), high blood pressure (3) and of the metabolic syndrome (4), factors that increase the risk of developing cardiovascular disease in adulthood, the leading cause of death in the world (5). Although in children there is no precise definition of metabolic syndrome due to the lack of agreement of scientific societies on the cut off values of individual parameters, it is accepted that, even in childhood and adolescence, obesity is frequently associated with excess visceral adiposity, glucose metabolism disorders, increased blood pressure (BP) and triglycerides and decreased HDL cholesterol. It is believed that hyperinsulinism is the pathogenic mechanism underlying these alterations. Fatty liver is also frequently observed (6, 7). Generally, for developing excess weight it is necessary that the intake of calories exceeds the energy expenditure. Fructose, however, could have an additional role in the development of obesity and cardiometabolic alterations independent from the energy provided by the nutrient itself. In fact, fructose may induce metabolic alterations by stimulating the production of some substances such as uric acid, lactate, methylglyoxal, ceramide. In addition, compared to other sugars, it induces a much greater production of triglycerides and free fatty acids (FFA) leading to energy imbalance (8). Fructose also promotes a state of chronic inflammation through the release of pro-inflammatory cytokines, while it blocks others with a protective effect, such as adiponectin. Moreover, its interaction with the control systems of hunger and satiety, through the establishment of leptin resistance, induces an increase in caloric intake. Finally, fructose has been found to stimulate the secretion of vasopressin (8). The knowledge of these specific effects of fructose should direct physicians toward using preventive strategies aimed at limiting its consumption, especially in childhood. Newborn children already show an innate predilection for the sweet taste and fructose is the natural substance that arouses this sensation the most (9). In the light of new findings on the metabolic role of fructose, the current high consumption of free sugars, especially by children and adolescents, should be of great concern. This review will specifically focus on the changes in clinical variables associated with fructose consumption and the role of fructose in the early development of cardiometabolic risk factors, with the aim of increasing the awareness of these changes in the medical community, particularly pediatricians, and all health care providers dealing with children.

## Sources of Fructose

The term “simple sugars” refers to the monosaccharides glucose, fructose and galactose and the disaccharides sucrose, lactose and maltose. From a nutritional point of view, the definition suggested by the World Health Organization (WHO) of “free sugars” excludes intrinsic sugars present in foods, such as lactose in milk and fructose in fruits, whereas it includes sugars added during the industrial or home preparation of foods and beverages. Free sugars also include those contained in honey, fruit juices and nectars (10). The free sugars that are present in food are glucose, fructose and sucrose. The sucrose molecule is composed of glucose and fructose and is broken down in the intestinal lumen by the disaccharidase enzyme. For this reason, the free sugars absorbed by the intestine are glucose and fructose. Fructose can be taken as a nutrient or it can be generated in the body through the polyol pathway which allows the transformation of glucose into fructose, through the intermediate compound sorbitol, by the enzymes aldose reductase and sorbitol dideoxygenase (11). This metabolic pathway, which is activated under conditions of physical or chemical stress, is especially important in some mammals that live in conditions of extreme temperature, lack of fluids and/or hypoxia, such as, for example, animals that hibernate (12, 13). In humans, the polyol pathway is activated in para-physiological or pathological states, such as decompensated diabetes, hyperuricemia, hyperosmolarity due to dehydration or excessive salt intake, oxidative stress, hypoxia or ischemia (13). However, in humans and especially in children, fructose excess is virtually always due to excessive intake (14). Fructose is contained in fruit, but in minimal percentages compared to the weight of the fruit itself. On the other hand, fructose is present in a high concentration in honey, it represents half of the content of sucrose (the normal table sugar) and reaches 55–60% in high fructose corn syrup (HFCS) abundantly used by the food industry, especially to sweeten soft drinks. Recently, the use of pure fructose, wrongly perceived by consumers as a natural product, has been gaining ground (Table 1). Most commercially available fructose is obtained by glucose isomerization of corn starch, with the same chemical process used to produce HFCS. Soft drinks, energy drinks, fruit juices and nectars, and generally free sugars in liquid form represent the main source of fructose consumed by children and adolescents. Even children in the first year of life can be exposed to an excessive intake of fructose, especially through the consumption of homogenized fruit. In many of these products, concentrated fruit juices made from high-fructose syrups are added. In this way it is possible to add the misleading claim “contains only fruit sugars” on the label.

**Table 1:** Dietary sources and endogenous production of fructose.

**Dietary sources (fructose content)**
Fruit (depending on ripening degree): Oranges (3.2%), bananas (5.2%), strawberries (2.3%), tangerines (2.8%), apples (8%), pears (6%), lemon (0.9%) Vegetables: Carrots (2.3%), lettuce (0.3%), eggplant (1.4%), bell peppers (1.5%), peas (0.5%), tomatoes (1.7%), zucchini (1%),
Honey (40%)
Sucrose (50%)
High Fructose Corn Syrup (55%, industrial production of sugar-sweetened beverages, cakes, baked goods, pastries, catch-up sauces)
Concentrated juice, e.g., some homogenized fruits (over 60%)
Fructose as sweetener (100%)
Fruit juice, palm sugar, maple syrup (variable amounts)
**Main conditions for endogenous fructose production from glucose through activation of the polyol pathway [ref. (11–13)]**
Hyperglycaemia (decompensated diabetes mellitus)
Hyperuricemia
Heat stress
Oxidative stress
Hyperosmolarity (dehydration or dietary high salt content)
Hypoxia
Ischemia

## Consumption of Free Sugars and Fructose

At the end of the 1700s, per capita sugar consumption in the English population was about 2 kg per year, and throughout the 1800s it did not increase much. Subsequently there was a dramatic increase in the use of this nutrient, reaching about 70 kg/year in 2000 in the US. Parallel to this phenomenon the prevalence of obesity and type 2 diabetes increased dramatically (14). As already mentioned, the greatest contribution to the excess of free sugars in the diet is accounted for by beverages that are sweetened with high-fructose syrups (15). It has been estimated that in the year 2000 the consumption of sugar-sweetened beverages (SSBs) in the US was 500 ml per person per day, about 190 liters per year, with a 5-fold increase from the 1950s to 2000 (16). In children, the introduction of free sugars into the diet starts very early, reaching high levels already in the first 3 years of life (17). However, pre-adolescence and adolescence are characterized by the highest consumption of free sugars (18, 19). Even in this age group, the greatest contribution to the intake of free sugars is given by the consumption of sugar-sweetened beverages. In fact, for many children, the limit of 10% of daily calories from simple sugars recommended by the WHO is exceeded solely by the consumption of SSBs (20). Available data are not sufficient to evaluate the exact amount of fructose, by disaggregating it from the total amount of sugars consumed, but it is reasonable to think that fructose constitutes at least 50% of the total intake of free sugars. Some information is provided by two American surveys carried out at a certain distance of time, the first referring to the years 1977–1978 (21) and the second considering the years 1999–2004 (14). When comparing these two surveys it can be seen that, in the time lapse between the two surveys, the consumption of fructose naturally present in food (mainly in fruit) has undergone a sharp decrease, while the consumption of added fructose tripled for all age groups. In childhood and adolescence, fructose consumption is much higher than in adulthood. In fact, the average consumption of added fructose in male teenagers is 68 g per day, about twice that of adults (14).

## Fructose Metabolism

**Figure 1:**
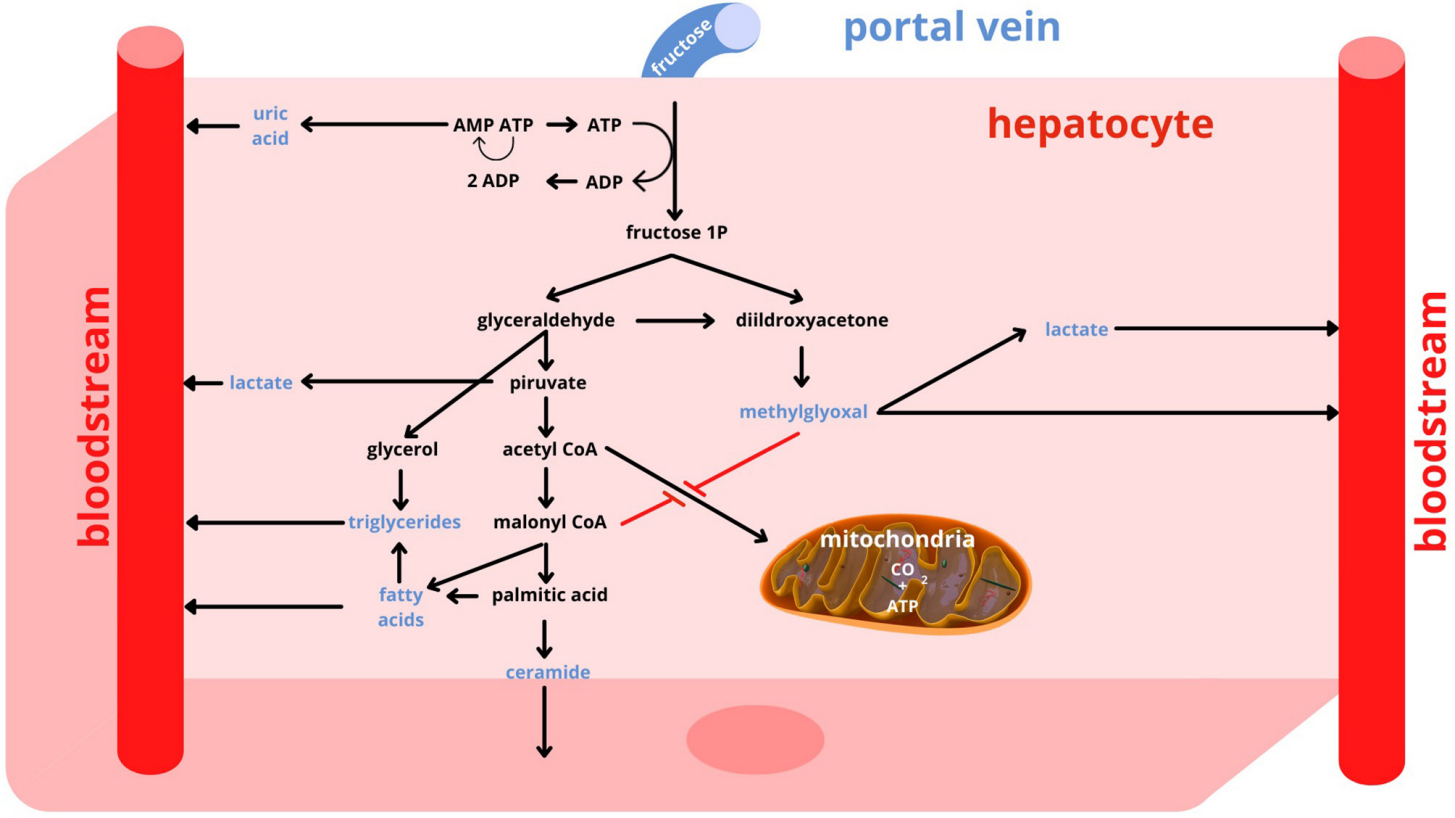
Fructose metabolism. AMP, adenosine monophosphate; ADP, adenosine diphosphate; ATP, adenosine trisphosphate; CO_2_, Carbon Dioxide.

The glucose transporter 5 (GLUT5) allows the uptake of dietary fructose through the brush border of the enterocyte, while the glucose transporter 2 (GLUT2) promotes its release from the basal pole of the cell into the systemic circulation, toward the portal vein (22, 23). The liver is the key organ for fructose metabolism. In fact, about 80% of dietary fructose is readily absorbed by the liver and only a small portion reaches other tissues (22). In addition to hepatocytes, only enterocytes, renal tubule cells and some cells of the central nervous system possess the enzymes necessary for the metabolization of fructose and the utilization of its metabolites (24). In contrast to glycemia, which increases strongly after meals intake and needs a long time to return to pre-prandial levels, fructosemia increases only slightly even after meals that are rich in free sugars, because the shift of fructose from the portal vein to the liver is very rapid. In addition, in the hepatocyte the metabolic pathways of fructose are uncontrolled, whereas glucose has a metabolism that is carefully regulated by mechanisms that depend on the energy level of the cell and the need to maintain glycemia in a normal range (25). The uncontrolled entry of fructose into the hepatocyte is a crucial point for the establishment of metabolic alterations that accompany an excessive and time-concentrated intake of this nutrient (Figure 1). In the hepatocyte, fructose is processed by the enzyme phosphofructokinase with adenosine trisphosphate (ATP) consumption. Excessive fructose intake therefore leads to a decrease in the energy level of the cell. Moreover, the degradation of ATP produces inosine monophosphate from which uric acid is formed. Uric acid on the one hand stimulates phosphofructokinase, and on the other hand flows into the circulatory system. Once phosphorylated, fructose is broken by the enzyme aldolase into two trioses: glyceraldehyde-3-phosphate and dihydroxyacetone phosphate. From the first compound pyruvate is generated, which is partially transformed into lactate that leaves the hepatocyte, passes into the systemic circulation and makes the uptake of glucose by muscle cells more difficult, thus increasing insulin resistance (26). Pyruvate also gives rise to the formation of acetylCoA that, in the presence of high levels of fructose and uric acid, tends to be involved in fatty acid production, rather than being used in mitochondria to produce energy. In case of an excessive intake of fructose there is an increased production of FFA that, when secreted as FFA, contribute to induce insulin resistance, while, when combined with glycerol, are excreted by the liver as triglycerides. After a high-fructose meal, therefore, both uricemia and triglyceridemia increase (27, 28). In addition, some of the fatty acids produced may remain in the hepatocyte and contribute to the development of fatty liver (29). A side product of the synthesis of fatty acids, in particular of palmitic acid, is ceramide, which also favors the establishment of insulin resistance (30, 31). Another important alteration induced by a high level of fructose is the production of methylglyoxal derived from dihydroxyacetone phosphate (32–34). Methylglyoxal is partly detoxified through the production of lactate, but it also has the effect of further reducing the passage of acetylCoA into the mitochondria. Thus, energy production tends to favor extra-mitochondrial anaerobic mechanisms over mitochondrial aerobic ones. Methylglyoxal also has an inhibitory action on the enzyme adenosine monophosphate kinase (AMPK). Inhibition of AMPK by methylglyoxal promotes a reduction in energy-producing catabolic pathways, such as glycolysis and fatty acid beta-oxidation, while activating energy-consuming anabolic pathways such as gluconeogenesis, cholesterol and fatty acid synthesis with release of glucose and very low-density lipoprotein cholesterol (VLDL) into the circulation. The release of fructose-derived glucose into the bloodstream via gluconeogenesis results in an increased demand for insulin. The energy intake accounted for by fructose is only slightly lower than that of glucose (25); fructose, however, cannot directly be used for energy purposes, but it can only indirectly through its transformation into glucose, fatty acids, and lactate with the production of a series of metabolites with potential negative effects. Overall, it can be considered that at the hepatic level about 50% of fructose is transformed into glucose, 15–20% into glycogen, 15–25% into lactic acid and the remainder into triglycerides, secreted as VLDL or stored in the liver (35).

## Role of Fructose in the Evolution

**Figure 2:**
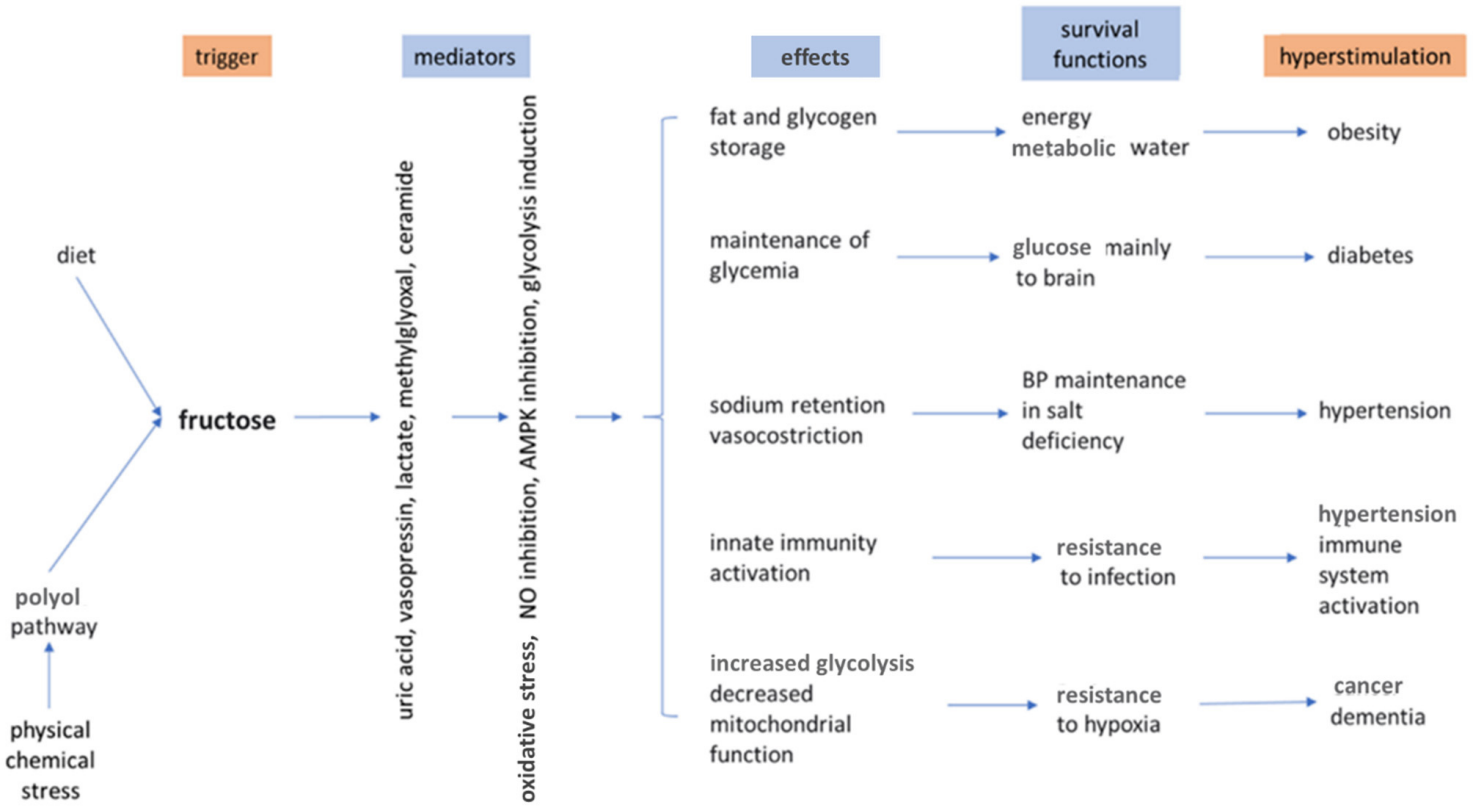
Effects of endogenous and dietary fructose. AMPK, Adenosine monophosphate kinase; NO, nitric oxide. Modified from Johnson et al. (13).

When studying the role of fructose in nutrition it is important to consider the different environmental conditions that have occurred during evolution. One of the evolutionary advantages is represented by the capacity to use the fructose present in fruits and, in smaller amounts, in vegetables, as an energy substrate. The predilection and receptors for sweet taste may have evolved to favor the use of sugars for energy production in herbivorous and omnivorous animals, but not in carnivorous ones. In addition to the use of fructose as an energy source, another evolutionary advantage has also been hypothesized. Fructose may be a kind of trigger activator of metabolic changes that may be advantageous in particular environmental conditions, such as climate change, famine, drought, conditions which only some species were able to overcome thanks to the ability to adapt, directing the metabolism to save resources and energy (13, 36). Bears, for example, prepare for hibernation by consuming ripe fruit and honey, thus taking a large amount of fructose that, directly or through its metabolites, activates a series of metabolic changes to ensure their survival during hibernation: the storage of fat and glycogen, the maintenance of a level of blood sugar and BP sufficient for vital functions and the decrease of oxygen consumption (37). During hibernation, the oxidation of stored fatty acids and glycogen by fructose supply is also an important source of metabolic water. Such metabolic changes, that are certainly useful to hibernating animals, can lead to a series of negative consequences if they occur in a completely different environmental situation, such as the current one of our species. Today, the majority of humanity lives in a non-hostile environment, which requires less energy expenditure and has many food resources available (38). In this context, the consumption of sugar, and in particular fructose, on the one hand stimulates the organism to save energy and resources, whereas on the other hand contributes to an excessive caloric intake. Thus, the metabolic changes induced by fructose that in the past helped our species to survive environmental stress, today favor the current epidemic of obesity and related diseases. In this scenario, some additional considerations must be made concerning uric acid (39). In almost all animal species uric acid is an intermediate metabolite of the purine catabolism, which is degraded by the enzyme uricase to allantoin, a water-soluble substance that is easily eliminated by the kidney. Only in humans and higher apes uric acid is present in biological fluids, because these species have lost the activity of the uricase enzyme (40, 41). Because increased uric acid has been described to be a risk factor for hypertension and mortality from cardiovascular causes (42), it seems odd that this particular mutation was selected and transmitted during evolution (43). However, in our ancestors a higher level in uricemia probably had favorable effects. In fact, somewhat higher values of blood glucose and BP were useful for fight or flight, while increased insulinemia favored fat storage that could be useful in times of low food availability. Finally, uric acid may also have had positive effects on brain function and immunity (36, 39). Thus, fructose, through the production of uric acid, may have aided the survival of species that had lost uricase activity. However, this mechanism is now probably negative in our current environmental situation, inducing an increase in the prevalence of obesity and non-communicable diseases. Figure 2 shows the positive and negative effects of endogenous and dietary fructose and the mechanisms by which these are mediated. Table 2 summarizes the main effects of a high fructose dietary intake on the most important organs.

**Table 2:** Effects of a high fructose dietary intake on major organs.

**Organs**	**Mediators**	**Metabolic effects**	**Clinical manifestation**
Adipose tissue	FFA UA	↑ROS ↑Inflammatory cytokines ↑FFA uptake ↑Lipid accumulation ↑Adiponectin secretion ↑Autophagy ↓Insulin sensitivity↓Glucose uptake ↓Leptin sensitivity ↓Oxygen availability	Inflammation Endothelial dysfunction
Brain	FFA UA MG	↑ROS ↑Inflammation cytokine ↓Insulin sensitivity ↓Leptin sensitivity ↑Food intake	Increase of appetite Psychological stress
Heart/vessels	FFA UA	↑ROS ↑ FFA uptake ↑Vascular tone ↓Vascular vasodilation ↑Blood pressure ↓Insulin sensitivity ↓Glucose consumption ↑Advanced glycation end products	Plaque formation Hypertrophy Endothelial dysfunction Vascular stiffness
Intestine	UA	↑Endotoxins ↑Bacterial composition disturbance ↑Dysregulation of tight junction protein ↓Insulin sensitivity	Increased intestinal permeability
Kidney	UA MG	↑ROS ↑Inflammatory cytokines ↑Dysregulation of renal organic ion transporters ↑Urine sodium retention ↑NO production ↓Insulin sensitivity ↓UA clearance	CKD Endothelial dysfunction
Liver	Lactate FFA Ceramide UA MG	↑Gluconeogenesis ↑Glucose export ↑*De novo* lipogenesis ↑ROS ↑Inflammatory cytokines ↑Lipid accumulation ↑VLDL-secretion ↑Mitochondrial dysfunction ↓Insulin sensitivity ↓Glucose consumption ↓Glucose uptake ↓Oxygen availability	Steatosis NAFLD Fibrogenesis Endothelial dysfunction
Pancreatic islet	Glucose FFA UA	↑Inflammatory cytokines↑Apoptosis ↑Endoplasmic reticulum stress ↓Insulin sensitivity ↓Leptin sensitivity	Glucose intolerance Increased β-cell mass Irregular insulin secretion
Skeletal muscle	Lactate FFA Ceramide UA	↑ROS ↑Inflammatory cytokines ↑FFA uptake ↑Lipid accumulation ↑Autophagy ↓Insulin sensitivity ↓Glucose uptake ↓Oxygen availability	Inflammation response Endothelial dysfunction

*CKD, Chronic Kidney Disease; FFA, free fatty acid; MG, methylglyoxal; NAFLD, non-alcoholic fatty liver disease; ROS, reactive oxygen species; UA, uric acid; VLDL, Very Low-Density Lipoprotein*.

*Modified from Zhang et al. (8)*.

## Fructose, Pregnancy, and Lactation

The term epigenetics refers to the science that studies the influence of the environment on the genetic expression, therefore on the phenotype. The human organism, from conception to the first 2 years after birth, is particularly sensitive to environmental stimuli. This period of life is called “The First Thousand Days” and the Developmental Origins of Health and Diseases (DPHaD) concept defines the programming of the metabolic and clinical fate of each individual. Epigenetic modifications affect not only the first offspring, but can also be transmitted to subsequent generations. In this context, the diet of the mother during gestation and lactation is particularly important, both quantitatively and qualitatively. In particular, the excess of free sugars and especially fructose, often present in western diets, may raise concerns. Even if further confirmations are needed, the results of some studies suggest pregnant women and nursing mothers to limit these nutrients.

It has long been known that fructose crosses the placenta (44) by diffusion (45), and probably also by an active transport mechanism (46). In addition, the feto-placental unit appears to be able to produce fructose from glucose via the polyol cycle (47, 48). Thus, fructose has a role in fetal metabolism. The intake of free sugars and fructose by pregnant women and nursing mothers is high (49). A diet that is rich in fruit has been associated with a decreased risk of pre-eclampsia (50) but, by contrast, excessive consumption of SSBs has been associated with an increased risk of pre-eclampsia and preterm delivery (51, 52). Several studies have been conducted in animal models in which varying amounts of fructose were administered during pregnancy. In some cases, the amount of dietary fructose was very high, resulting in an excessive intake of calories and limiting that of other important nutrients. This makes it difficult to interpret the results, and particularly to distinguish the direct effect of fructose from that of excess calories and the presence of other nutritional deficiencies. The studies performed in animal models have shown that, compared to control groups, high fructose intake was associated with a number of metabolic changes including: 1. insulin resistance, glucose intolerance, pregnancy diabetes and fatty liver in mothers (53–55); 2. decreased weight and vascularization of the placenta (56); 3. hyperglycemia, hyperinsulinemia and hypertriglyceridemia in fetuses (55); and 4. reduced birth weight (57), increased body weight, fat mass and lipid profile in offspring (26, 58, 59). In addition, a decreased sensitivity to leptin has been demonstrated in both mothers and fetuses (60). Regarding blood pressure, several studies show an increase in blood pressure values in adult animals born from mothers fed with high amounts of fructose during pregnancy (61–63). Among these, the work of Seong et al. (64) is particularly interesting, as it does not only report an increase in systolic blood pressure and angiotensin II compared to controls in the first and second generation of mice born from mothers fed a diet with 20% fructose, but also an increase in renin and aldosterone found until the third and fourth generation, respectively.

Data from animal models in which fructose was administered only during lactation and not also during gestation are scarce. Alzamendi et al. (59) reported an increase in weight, food intake and decreased leptin sensitivity in male rats fed by mothers on 10% fructose diets. Similar effects were found in rats fed a fructose-rich formula milk (65). In a very interesting study by Goran et al. (66) the presence of fructose in human milk and in children fed exclusively with breast milk until the sixth month was analyzed. In breast milk the concentration of fructose was very low, equal to about 7 μg/mL, which is more than 30 times less than the concentration of glucose. Despite his low quantity, fructose is the only sugar that is associated with significant changes in the body composition of infants. Indeed, it is significantly associated with body weight, lean mass, fat mass, and bone mineral content assessed at the age of 6 months. Although the authors point out that their data are not sufficient to demonstrate a causal relationship, since it is not precisely known how sensitive such young children are to fructose, it is important to state that it would be appropriate to pay attention to the consumption of free sugars not only during gestation, but also during lactation.

## Fructose and Cardiometabolic Risk Factors in Pediatric Age: Pathophysiological And Clinical Aspects

The analysis of the pathophysiological mechanisms that link an excessive intake of fructose to the development of arterial hypertension and other cardiometabolic risk factors is not easy for a number of reasons. First, the amount of fructose to be considered “excessive” is not well defined. Generally speaking, the consumption of fructose in fruits and vegetables should be encouraged because, thanks to the presence of other micronutrients, it is believed not to have the same negative effects as when it is taken as free sugar. The indication of the WHO not to exceed an intake of free sugars equal to 10% of daily calories is agreeable, but the specific quantity of fructose, the form in which it is taken (solid foods or beverages) and the time space in which it is taken should always be considered. Second, available data from studies in children are indirect and refer to fructose consumption in the form of SSBs or sweeteners added to foods. However, it should be emphasized that in these studies, fructose constitutes only one of the sweeteners used and is often added in association with glucose. Third, it is impossible to separate the direct effects of fructose from those of its metabolites. In children, fructose consumption is the main cause of increased uricemia. However, uric acid may also be generated by other sources, either endogenous (purine catabolism and some amino acids) or dietary (glutamate, purines, uric acid) and uricemia is influenced by genetically determined factors such as renal and intestinal excretion of uric acid. The metabolic and cardiovascular effects of uric acid should be discussed separately, however, and such an issue is not among the aims of this review. Some adverse effects of fructose on various organs and apparatuses have been reported, but a clear pathogenetic explanation has not yet been described. Finally, the various cardiometabolic risk factors influence and potentiate each other. For example, hyperinsulinism promotes the development of visceral obesity, which, in turn, promotes the establishment of insulin resistance. In the following paragraphs, we will briefly describe the relationship between fructose consumption and different cardiometabolic risk factors. A larger space is reserved for the relationship between fructose and the development of arterial hypertension, particularly in pediatric age.

### Obesity

Animal studies have provided information about the specific role of fructose in the development of obesity (67, 68). In addition to simply inducing calorie intake, fructose contributes to the development of excess weight through several additional mechanisms. Some of them have already been described, among which the *ex novo* production of fatty acids and triglycerides and the reduction of substrate oxidation at mitochondrial level. In addition, several metabolites of fructose (lactate, FFA, ceramide) contribute to the establishment of insulin resistance and the increase of insulin, in turn, promotes the deposition of adipose tissue. Fructose, through mechanisms not yet fully understood, may also be able to induce a resistance to the action of leptin on the satiety center in the brain, leading to an increase in food intake and the development of a positive caloric balance (11, 69–71). Fructose also has other actions that promote food intake, such as the inhibitory effect on the expression of YY peptide, Y neuropeptide, and propiomelanocortin, and the stimulation of cannabinoid 1 production (72). Finally, through mechanisms involving the development of reactive oxygen species (ROS) and activation of the renin-angiotensin system, fructose promotes the genesis of visceral white adipose tissue, while reducing thermogenesis produced by brown adipose tissue (73). In addition, uric acid *per se* promotes insulin resistance and induces a state of chronic inflammation. Many epidemiological studies have demonstrated an association between the consumption of free sugars and the development of obesity, particularly in children and adolescents. A meta-analysis of cohort studies found that a higher intake of SSBs among children was associated with a 55% higher risk of being overweight or obese compared to those with a lower intake (74). A study performed in a population of preschool-aged children found that the prevalence of obesity at 4.5 years of age was more than twice as high in sugar-sweetened beverage drinkers as in those who did not consume any (75). Children aged 3–6 years with overweight mothers during pregnancy, consumed more SSBs (but less milk) and had higher waist circumference than peers born to mothers with normal body mass index (BMI) (76). It has been shown that in developed countries, lower socioeconomic classes have the highest prevalence of excess weight in pediatric age. In a low-income African-American preschool population of children aged 3–5 years, SSB consumption correlated positively with the presence of overweight and obesity at baseline, and worsening of weight class was more pronounced in more frequently SSB users over a 2-year follow-up (77). A prospective Singaporean study observed that the consumption of SSBs in infants (18 months) was not associated with adiposity measures (BMI and skinfold thickness) and excess weight at 6 years of age. In contrast, in preschool children SSB intake was associated with higher body weight, skinfold thickness and risk of overweight/obesity at age 6 years (78). A German study suggested that higher consumption of free sugars during the first year of life does not lead to an increase in BMI values at 7 years, whereas this happens in subjects with higher free sugar intake in the second year of life (79). Regarding adolescents, an American study described a relationship between consumption of SSBs and increased body weight in a sample of more than 10,000 boys aged 9–14 years. According to the authors, this would be mainly due to the additional energy intake provided by these drinks. In fact, the effect appears attenuated as calories are reduced (80). On the other hand, in a large population-representative birth cohort of Hong Kong Chinese children, frequency of SSB consumption at 11 or 13 years was not associated with subsequent BMI z-score or overweight (including obesity) up to 18 years, nor with waist circumference and waist to height ratio at 16–19 years. This may be due to a much lower SSB intake of the participants of the study (81).

### Disorders of Glucose Metabolism and Type 2 Diabetes

Glucose and fructose, besides contributing to caloric excess, alter the glycide metabolism through different mechanisms. Glucose determines a high glycemic load and glucotoxicity can damage pancreatic beta cells (82), while fructose has little effect on glycemia, but promotes the development of insulin resistance. In experimental animals, fructose has been shown to reduce insulin receptor sensitivity (83). Moreover, fructose contributes to the increase of FFA production by the liver (84). FFA appear to decrease the sensitivity of skeletal muscle to insulin (85). Moreover, FFA (saturated and long chain), through the production of ceramide, have a lipotoxic effect on pancreatic beta cells that in time may favor the development of diabetes (86), especially in genetically predisposed subjects. High levels of fructose have also been found to determine a reduction in the production of adiponectin with a consequent increase in insulin resistance (87). Finally, both glucose and fructose promote the glycation of proteins and amino acids with the resulting production of advanced glycation end products (AGEs) that also contribute to the development of diabetes (88). The progression of insulin resistance and the consequent worsening of glycemic control have a negative effect on the residual function of pancreatic beta cells and contribute to the production of AGEs (89). Uric acid plays a role in glucose metabolism as well, especially by contributing to insulin resistance (90). Between 2001 and 2017, there has been a considerable increase in the prevalence of type 2 diabetes mellitus in children and adolescents (from 0.34 in 2001 to 0.67 in 2017, per 1,000 subjects in this age group) (91). It is very likely that changes in the eating habits of children and particularly the increased consumption of SSBs are largely responsible for this situation. Unfortunately, no studies are available that correlate the consumption of fructose, free sugars or SSBs during childhood with the development of type 2 diabetes or other alterations in glucose metabolism in adulthood. However, results from studies in adults make this possible correlation very likely. The UK Scientific Advisory Committee on Nutrition concludes that, in adults, there is consistent evidence that consumption of SSBs is associated with an increased risk of developing type 2 diabetes (92). Similar conclusions are reached by two meta-analyses performed on adult populations (93, 94). Another meta-analysis in adults showed that an increase in consumption of SSBs by one serving per day was associated with a 13% greater incidence of type 2 diabetes, even after correction for excess weight (95). On the contrary, it has been shown that a correct diet that required the decrease/abolition of SSB consumption reduced the relative risk of developing type 2 diabetes (96). Finally, in a population of young women, a high glycemic index diet was shown to be correlated with the development of type 2 diabetes (97).

### Dyslipidemia

Fructose is the most important lipogenic sugar and, as described before, a large proportion of dietary fructose intake is converted to lipids through *ex novo* synthesis of fatty acids. This process is increased in the presence of chronic fructose consumption, which not only increases intestinal reabsorption of fructose, thanks to the synthesis of GLUT5 transporters (98), but also induces an increase in the expression of all hepatic enzymes involved in lipid synthesis. In fact, fructose stimulates the expression of the sterol regulatory element-binding protein 1c (99) and of carbohydrate-responsive element-binding proteins (100–102) that, together with other cofactors such as peroxisome proliferator-activated receptor gamma coactivator 1-beta (103), are the main regulators/stimulators of lipid synthesis (104). Since insulin regulates the synthesis of these element-binding proteins too, fructose can indirectly stimulate their expression also by increasing insulin levels through its effect on insulin resistance. Insulin excess also stimulates the secretion of ApoC-III, a substance able to decrease the activity of lipoprotein lipase and the hepatic clearance of lipoprotein remnants, with a consequent further increase in plasma levels of triglycerides and lipoprotein remnants, substances that are particularly atherogenic (105). Overweight children and adolescents more frequently have dyslipidemia than those of normal weight (106). By contributing to the development of obesity, fructose indirectly contributes to the establishment of various forms of dyslipidemia. However, a meta-analysis (107) has shown that in adults the increase in total cholesterol and LDL-cholesterol is determined only by the intake of very high amounts of fructose (108) and, according to some authors, only if associated with a simultaneous consumption of glucose or excessive caloric intake (109). The intake of high doses of fructose does not seem to induce any reduction in HDL-cholesterol that, together with the increase of triglycerides, is one of the characteristics of the dyslipidemia observed in the metabolic syndrome (107). Not many studies are available in children that associate cholesterol levels with fructose consumption. It has been described that obese children who consumed higher amounts of fructose had higher triglyceride values and lower HDL-cholesterol values than normal-weight peers, and fructose consumption correlated directly with the presence of smaller and therefore more atherogenic LDL particles (110). Interestingly, isocaloric fructose restriction resulted in a 46% decrease in LDL-cholesterol in obese children with metabolic syndrome (111). In contrast, an increase in total cholesterol was observed in severely obese children and adolescents who consumed higher amounts of fructose than a control group (112). In three studies performed in children and adolescents, it was observed that increased consumption of SSBs was associated with decreased HDL-cholesterol, but also with increased BMI values (113–115). Therefore, a direct relationship between fructose intake and plasma cholesterol values in pediatric age has not yet been completely clarified. On the other hand, in young adults an increase in triglycerides and VLDL is associated with both acute and chronic fructose consumption (28, 116, 117), even at relatively low doses (118). Regarding the hypothesis of an association between consumption of SSBs and triglycerides in pediatric age, data are conflicting: some studies show an increase in triglyceridemia in children and adolescents with higher consumption of SSBs (113, 115, 118), whereas others studies do not show any significant differences (119, 120).

### Non-alcoholic Fatty Liver Disease

The term NAFLD refers to a progressive combination of intrahepatic lipid accumulation (steatosis), inflammatory processes (steatohepatitis) and fibrosis (liver cirrhosis). In addition to the *ex novo* production of fatty acids and the decrease in their oxidation related to an excess intake of fructose, also the influx of circulating FFA and chylomicrons contributes to an increase in the intrahepatic lipid pool that accumulates and results in the development of NAFLD (121). Fructose consumption promotes inflammatory processes, either directly or via uric acid, by stimulating the generation of ROS and hepatotoxic glycation products. Inflammation, in turn, promotes fibrosis (122, 123). It has been observed that subjects affected by NAFLD have an increased expression of fructokinase and fatty acid synthase and a greater tendency to ATP depletion and therefore to the production of uric acid (124). Non-alcoholic fatty liver disease, although often associated with excess weight, can also occur in non-obese subjects, both adults and children, who consume high amounts of fructose as free sugar (125). A study performed on a population of children and adolescents with weight excess and NAFLD, in which fructose consumption and serum uric acid were assessed, showed a prevalence of non-alcoholic steatohepatitis (NASH) of 37.6%. Both serum uric acid concentration and fructose consumption were independently associated with NASH and fructose consumption was independently associated with hyperuricaemia. Subjects with NASH had higher waist circumference, transaminase, triglycerides, and TNF alpha levels. Thus, dietary fructose consumption and serum uric acid levels are interrelated and independently associated with the presence of early hepatic organ damage (126). The North American Society for Pediatric Gastroenterology, Hepatology and Nutrition guidelines highlight the higher prevalence of NAFLD in obese children compared with children with normal weight, but also report that not all children with NAFLD are obese. Indeed, the at-risk population suggested to be screened for NAFLD includes both obese and overweight children with cardiometabolic risk factors (insulin resistance, prediabetes, diabetes, dyslipidemia, and central adiposity) (127). A pilot study performed in a numerically small population of children with NAFLD, demonstrated that a low-fructose diet was able to induce an improvement in oxidized LDL levels, a marker of cardiovascular disease (128). Finally, 24 overweight Hispanic-American adolescent consumers of sweet drinks with hepatic steatosis were enrolled and randomized to drinking beverages that contained either fructose only or glucose only. After 4 weeks, insulin sensitivity, inflammatory status, plasma FFA, and LDL oxidation had improved in the group of patients taking the glucose-sweetened drinks. Although regression of hepatic steatosis was not observed, the study showed that reducing dietary fructose leads to an overall improvement in cardiovascular risk factors, suggesting that this sugar plays a central role in increasing cardiovascular disease risk in NAFLD patients (129).

### Hypertension

Several decades ago it was already described that high fructose diets could induce experimental hypertension. Sprague Dawley rats, fed a diet in which fructose constituted more than 60% of the total caloric intake, developed systolic hypertension after 2 weeks and the same outcome was observed in dogs subjected to a similar diet (130, 131). In both studies, the increase in blood pressure was associated with an increase in insulinemia (insulin levels) and insulin resistance. The role of insulin resistance in the development of fructose-related hypertension was confirmed by the demonstration of a reduced density of insulin receptors in skeletal muscle and liver of rats with fructose-diet hypertension (132). Interesting data were also obtained more recently in genetically hypertensive rats, in which a high fructose diet induced a further increase in BP values as the animal aged, associated with an increase in the levels of reactive oxygen species and lipid peroxidation in the rostral ventrolateral medulla. The study suggests the presence of an interaction between predisposing genetic factors and dietary habits in the establishment of hypertension (133). The autonomic nervous system and the renin angiotensin aldosterone system also appear to play a role in experimental fructose hypertension. Involvement of the two systems in fructose hypertension was demonstrated in experimental studies by showing that both sympathectomy and drugs inhibiting the renin angiotensin aldosterone system (RAAS) activity were able to reduce both insulin resistance and high BP values due to high fructose diets (134, 135). Furthermore, a study performed in high fructose-fed rats showed that the establishment of hypertension was associated with an increase not only in insulin resistance, but also in urinary catecholamine excretion, alpha adrenergic receptor density, and angiotensin II content in the left ventricle. Administration of an AT1 receptor antagonist was able to reduce both catecholamine excretion and adrenergic receptors, without exerting any effect on insulin resistance (136). Moreover, in fructose-fed rats, treatment with an alpha-blocker drug prevented the increase in BP without affecting insulin levels and insulin sensitivity (137). Further evidence was recently provided by Chen et al. who demonstrated that in rats with fructose-induced hypertension, excessive fructose intake led to an increase in fructose concentration in the cerebrospinal fluid, a decrease of nitric oxide (NO) levels in the nucleus tractus solitarii and a reduction of baroreflex sensitivity. Taken together, these data suggest that fructose leads to sympathetic hyperactivity, which in turn results in the development of hypertension (138). A small randomized trial performed in young healthy volunteers (21–33 years old) that evaluated cardiovascular system responses to fructose ingestion confirmed the involvement of the NTS. Fructose ingestion caused an increase in both systolic and diastolic BP and in heart rate. Spectral analysis of heart rate variability (HRV) showed that fructose ingestion also caused an initial increase in the low frequency component (LF, broadly taken as an expression of sympathetic activity) of HRV, and a subsequent reduction in the high frequency component (HF, expression of vagal activity), suggesting a significant reduction in baroceptor sensitivity (139). More recently, in a large sample of children aged 11/12 years, it was shown that high intake of SSBs was associated with increased values of systolic and diastolic blood pressure and with a shortening of the pre-ejection period of the cardiac cycle, considered a marker of sympathetic activity. The result was independent of the children's weight class (140). Some authors have suggested an association between fructose consumption and endothelial dysfunction. Endothelial dysfunction, which is characterized by a prevalence of vasoconstrictors over vasodilators, may determine an increase in BP values. It has been demonstrated that fructose-related hypertension (68, 141) is associated with an inhibition of the vasodilator effect of prostacyclin and acetylcholine (142, 143) and with an enhancement of the action of vasoconstrictor substances in experimental animals (144). A proper endothelial function is the result of the balance between the factors that promote vasodilation, NO and prostaglandins, and those that induce vasoconstriction, such as endothelin-1 (145). Oxidative stress and inflammation may play an important role as well (146). Fructose, either directly or through the action of uric acid, may interact with these processes. Nitric oxide is the most important dilating factor at the vascular level and is produced by the endothelium thanks to the catalyzing activity of the isoenzyme nitric oxide synthase (eNOS); the chemical reaction involves L-arginine and molecular oxygen and requires some cofactors, one of which is tetrahydrobiopterin (BH4). Alterations in NO production at the vascular level have been observed in several cardiovascular diseases, including hypertension (147). Fructose can interact with this system in several ways. Fructose-fed rats have been shown to exhibit increased activity of the enzyme L-argininase, which reduces the availability of L-arginine (148). In addition, an excess of fructose also has the effect of decreasing the availability of BH4 (149). In the absence of the substrate arginine and/or the cofactor BH4, eNOS promotes the development of superoxide anions from molecular oxygen that may interact with the same enzymes that produce BH4 (150). In fructose-fed rats, superoxide anions interact with other molecules and produce potent oxidants, which, in turn, impair the function of several proteins including eNOS, thus reducing the availability of NO and its vasodilator action (151). Over time, the reduction of eNOS activity may favor the proliferation of muscle tonic cells resulting in a non-reversible increase in vascular stiffness and BP values (152). Fructose-induced hyperinsulinism may also play a role in the development of arterial hypertension, either by directly reducing NOS production or by promoting the release of endothelin-1 (153, 154). Experimental animals fed a fructose-rich diet have a condition of oxidative stress, with higher levels of oxidant agents and reduced availability of antioxidant agents (155–157). Oxidative stress, in addition to decreasing NO activity, may contribute to endothelial dysfunction by increasing inflammation, tone, and by vascular remodeling (158). Whereas, no studies are available in children that correlate fructose as such taken *per os* with increased BP values, some evidence has been reported in young adult healthy volunteers (139). In most studies performed in populations of children and adolescents, only the relationship between general intake of free sugars, especially in liquid form as SSBs, and BP values was evaluated. From the results of these studies, it is difficult to evaluate the specific effect of fructose on BP, also because a high dietary intake of fructose generally leads to a worsening of weight status, a factor frequently associated with the presence of hypertension. For what regards the relationship between consumption of SSBs and BP, some meta-analyses, in adults (159, 160) and in children (161) confirm the tendency of BP values to increase with increasing consumption of SSBs. All studies, from different countries around the world, show that the consumption of drinks with added sugar is worryingly widespread among both children and adolescents. Several observational studies that involved a very large number of subjects aged between the second decade of life and the end of adolescence, assessed the intake of SSBs using food history questionnaires. Overall, the studies showed higher systolic blood pressure (SBP) values in subjects who consumed more of these products, but in most cases the increase in BP was accompanied by higher values of BMI or waist circumference (113, 114, 162). Only one study describes a higher prevalence of hypertension in normal-weight SSBs consumers than in overweight peers (163). Interesting are the results of a Brazilian study that confirms the presence of higher BP values in consumers of SSBs than in non-consumers; furthermore, this study shows that subjects that are accustomed to using low-calorie diet products show the highest BP values (164). A study on a large sample of Chinese boys reported an increase not only of SBP, but also of diastolic blood pressure (DBP) in heavy SSB users (115), while in another study the increase was observed only for SBP and in males (165). Not all studies confirm the finding of a positive association between hypertension and SSB consumption, however. In a Chinese study, no relationship between SSBs, obesity, and hypertension could be described (166), whereas in another study even an inverse relationship between SSB consumption and SBP values was observed (167). On the other hand, a prospective study evaluating SSB consumption in a population of 424 children and adolescents aged 6–18 years followed for 3.6 years described an incidence of elevated BP values (>90th percentile) almost three times higher in the quartile of subjects with the highest consumption of SSBs compared with those with the lowest consumption (119). Of particular interest is a recent intervention study that confirms the close relationship between SSBs and BP. The research was conducted in 30 overweight and obese adolescent boys, all heavy users of SSBs. The sample was randomized to take SBBs or an isocaloric amount of low-fat milk for 3 weeks. The study included a cross-over phase between the two groups after a washout period. The SBP z-score and uricemia were significantly lower when milk was taken instead of SSBs (120). Only one study evaluated the consumption of sugars taken via solid foods (sugary snacks) and confirmed that the subjects with the highest level of consumption had an increased risk of being hypertensive. It should be noted however, that these snacks also had a high salt content (168). Several studies showed that high salt intake is associated with increased BP values and hypertension prevalence in the pediatric population. It is interesting to note that a significant association between SSBs and salt intake was also described in children and adolescents (169). Table 3 shows the main available studies assessing the relationship between fructose intake and BP in pediatric age.

**Table 3:** Main available studies assessing the relationship between SSB intake and BP in pediatric age.

**References** **Country**	**Sample size (number of subjects)**	**Age range**	**Study design**	**Outcome**	**Main results**
Nguyen et al. (162) US	4,867	12–18	Cross-sectional study	SBP adjusted for age, race/ethnicity, sex, total calories, BMI z-score, sodium intake, smoking, and alcohol	Higher SSB consumption associated with higher serum uric acid (increased by 0.18 mg/dL) and SBP (increased by 0.17 z-score).
Bremer et al. (114) US	6,967	12–19	Cross-sectional study	SBP	Higher SSB consumption associated with higher HOMA-Index, waist circumference, BMI and SBP (High intake vs. low intake: 111.1 vs. 107.9 mmHg, *p* = 0.03).
Ambrosini et al. (113) Australia	1,433	14–17	Cross-sectional study	SBP and DBP adjusted for adjusted for age, pubertal stage, physical activity, dietary misreporting, maternal education, and family income.	Higher SSB consumption associated with higher SBP (highest tertile vs. lowest tertile +1.5 mmHg, *p* = 0.03) and overweight/obesity risk (OR: 4.8, 95%CI: 2.1–11.4).
Lin et al. (165) Taiwan	2,727	12–16	Cross-sectional study	SBP adjusted for study area, age, gender, physical activity, total calories, intake of meat, seafood, fruit, fried, food with jelly/honey, alcohol drinking, smoking.	Higher SSB consumption associated with higher SBP (highest SBB intake vs. no intake +3.47 mmHg, *p* = 0.004).
Mirmiran et al. (119) Iran	4,24	6–18	Prospective study	Incident hypertension.	Higher SSB consumption associated with higher hypertension incidence (highest quartile vs. lowest quartile: OR 2.79, 95%CI 1.02–7.64).
Asghari et al. (168) Iran	4,24	6–18	Prospective study	Incident hypertension adjusted for age, sex, total energy intake, physical activity, dietary fiber, family history of diabetes, and body mass index.	Higher energy-dense nutrient-poor solid snack intake associated with higher incidence of hypertension (OR: 2.99, 95%CI: 1.00–8.93).
Souza et al. (164) Brazil	488	9–16	Cross-sectional study	SBP and DBP adjusted for sex, age, BMI, physical activity, addition of salt to food, and education of the head of the family.	Higher soft drink consumption associated with higher SBP/DBP (no soft drink vs. SSB vs. diet soft drink: mean SBP 99.7 vs. 101.8 vs. 105.1 mmHg, *p* = 0.01 and mean DBP 57.2 vs. 58.2 vs. 60.5 mmHg, *p* = 0.04)
Gui et al. (166) China	53,151	6–17	National cross-sectional study	Prevalent hypertension adjusted for age, sex, residence, socioeconomic status, diet, screen time, and physical activity.	Neither general obesity nor hypertension associated with SSB consumption.
de Boer et al. (140) Netherlands	2,519 + 769	5–6/11–12	Cross-sectional study	SBP and DBP (adjusted for ethnicity, BMI, physical activity, screen time, gestational age, birth weight, maternal and paternal BMI, pubertal stage	Higher SSB consumption associated with higher SBP at 11–12 age (highest tertile vs. lowest tertile: SBP +2.3 mmHg, *p* = 0.006)
Qin et al. (163) China	10,091	9–12	Cross-sectional study	Prevalent hypertension adjusted for school, parental education, physical activity, diet intake.	Higher SSB consumption associated with higher hypertension prevalence (overall: OR1.40, 95%CI 1.15–1.70); normal weight: OR 1.78, 95% CI 1.20–2.65; overweight or obese: OR 1.28, 95% CI 1.01–1.61)
He et al. (115) China	2,032	7–18	Cross-sectional study	Prevalent hypertension.	SSB consumption associated with the risk of obesity (OR 2.08, 95% CI 1.21–3.54) and hypertriglyceridemia (OR 1.70, 95%CI 1.02–3.06), but not with a significant increase in the prevalence of hypertension.
Zhu et al. (167) China	3,958	6–17	Cross-sectional study	SBP (adjusted for age, sex, daily energy intake, pubertal stage, sedentary time, maternal education, and household income)	Higher SSB consumption inversely associated with SBP values (*p* < 0.05)
Perng et al. (170) Mexico	242	8–14	Cross-sectional study	SBP and DBP (adjusted for age and pubertal status)	Higher SSB intake associated with higher BP values (highest quartile vs. lowest quartile: SBP +4.65 mmHg and DBP +3.08 mmHg in girls, *p* = 0.07 and SBP +8.79 mmHg and DBP +7.1 mmHg in boys, *p* < 0.001).
Chiu et al. (120) US	30	13–18	Two-period randomized study (SBBs vs. low fat milk for 3 weeks with crossover to the alternate beverage after a ≥ 2 weeks washout)	SBP	SBP z-score (0.0 vs. 0.2, *p* = 0.04) and serum uric acid (362 vs. 381 umol/L, *p* = 0.02) significantly lower after milk compared to SSBs.

*BMI, body mass index; DBP, diastolic blood pressure; SBP, systolic blood pressure; SSB, sugar-sweetened beverage*.

## Intervention Directed at the Youth Consumption of Sugar Sweetened Beverages

Consumption of sweetened beverages, such as non-diet soft drinks, regular soda, iced tea, sports drinks, energy drinks, fruit punches, sweetened waters, and sweetened tea and coffee, is considered an important contributor to the widespread diffusion of obesity and its complications. As noted above, when considering studies related to the effects of excessive intake of SSBs, however, it should be emphasized that we are not referring to the intake of fructose alone, because glucose is also contained in these beverages. The evidence of the association between excessive SSBs and excess weight has raised the question of how to reduce their consumption in children and adolescents. Many scientific societies called for reducing the consumption of sugary drinks in pediatric age by fixing, in different ways, the daily quota not to be exceeded. However, it should be noted that these indications are only mediations to mitigate the current exaggerated hedonistic consumption of free sugars and do not correspond to the real needs of the human body. In fact, the daily consumption of free sugars could be equal to zero, as the need of glucose can be entirely satisfied by complex carbohydrates with low glycemic index, while a daily consumption of fruits and vegetables is sufficient to meet the need of fructose. Interventions aimed at reducing the consumption of SSBs in children include both educational interventions, carried out with the aim of improving eating habits, and environmental interventions to reduce the availability of SSBs. A typical example of the latter is the proposal of a “sugar tax.”

Since children spend most of their day time in school, this is an ideal setting for programs targeting healthy dietary behaviors (171). School-based intervention programs have been shown to yield positive results in preventing and reducing obesity (172). Many interventions have been performed in schools to see if educational programs with the purpose of reducing SSB consumption had an effect on body weight and the prevalence of excess weight in groups of children and adolescents (173). Considering only the randomized trials, altogether the results were positive, albeit with some differences. The association between consumption of SSBs and excess weight has been unequivocally demonstrated by a study performed in normal-weight children aged 5–12 years. Participants were randomized to receive once a day 250 mL of a beverage sweetened with non-caloric substances (sugar-free group) or a beverage sweetened with sucrose that provided 104 kcal (sugar group). At the end of an 18-month follow-up, the sugar group had higher values of body weight, BMI z-score, skinfold-thickness, waist-to-height ratio, and fat mass than the sugar-free group (174). A trial that randomized 644 children aged 7–14 years to receive an educational intervention resulted only in a modest reduction in SSB consumption in the intervention group compared with the control group, that was, however, associated with a significant reduction in the prevalence of excess weight (175).

A pilot study, also aimed at examining the effect of decreasing SSB consumption on body weight, did not lead to a significant reduction in BMI in the intervention group, despite the fact that in this group SSB consumption was almost completely eliminated. However, a significant reduction in BMI was observed among subjects with the highest body weight values at baseline (176). Another randomized trial performed in 7–9 year old students was targeted to reduce the availability of SSBs and increase the availability of water. The trial did not impact the SSB consumption of the entire study population, however and a positive result was observed in girls only (177). Finally, in another study, 224 obese adolescents who were regular consumers of SSBs were randomly divided into two groups, one in which a 1-year intervention was planned, designed to decrease consumption of sugar-sweetened beverages and a control group that did not include any intervention. In the first year of follow-up, consumption of SSBs in the intervention group was significantly lower than in the control group and remained so in the second year of observation. BMI values were significantly lower in the intervention group, but this difference disappeared at the end of the second year of follow-up (178). In spite of these results, which emphasize the need of additional intervention studies on this issue, a systematic review by Frank Hu et al. concluded that, overall, there is enough scientific evidence to support the possibility to reduce the prevalence of excess weight in children by decreasing consumption of SSBs (179). The need to reduce consumption of SSBs by children and, especially, adolescents led many countries to impose taxation on these beverages to discourage their consumption (180). The purchase of SSBs should not be discouraged only among adolescents, but also among adults, who are role models for youth and buy most of the SSBs consumed by children (181). In many countries of the world the sugar tax, in different forms, has been introduced since several years (182). One of the first pioneer countries to introduce a sugar tax was Denmark in 1930. The United Kingdom (UK) has introduced the SSB tax in 2018. In the US taxes have been imposed mostly on sodas and were never >10% of the price (183). Increasing taxation of SSBs and subsidizing healthy drinks could impact the reduction of obesity in children and adolescents (184). Current findings in the literature are not very strong, but suggest potential benefits regarding dietary behaviors from the implementation of such a tax (185). Several studies examining the effect of taxes on young school children or adolescents found limited effects on SSB consumption or BMI (186–188). More recent studies have provided more encouraging results. A US trial assessed if taxes could influence sweetened beverage intake in 86 928 high school students and found that the tax was associated with a statistically significant reduction in SSB consumption of 0.81 servings per week, 2 years after the implementation (189). A Mexican study assessed whether increasing the price of SSBs was associated with adolescent weight-related outcomes after implementation of the SSB tax. Increased prices were associated with decreased prevalence of excess weight among girls only, mostly in those with higher BMI, and where the price increment due to the tax was greater than 10% (190). The fact that a higher tax may be more effective has also been suggested by a qualitative study performed in a Detroit suburban middle school that gathered information on how a 20% tax on SSBs would affect adolescent SSB consumption. Most students reported that they would decrease their consumption of SSBs if a 20% tax were implemented (191). In conclusion, a sugar tax combined with adequate information campaigns on the health harms of SSBs and a reduction of sugar content in recipes by companies could have a significant impact for healthy eating habits in children and adolescents in the coming years. Moreover, additional studies testing the efficacy of interventions aimed at reducing fructose and free sugars intake in children and adolescents are needed.

## Conclusions

Despite the fact that in recent years the attention of parents to the eating habits of their children has increased, there still may be not enough concern about the intake of free sugars and fructose, as these nutrients may be erroneously perceived as “natural” and “necessary” and are therefore proposed even to the youngest children. Free sugars, however, are not at all indispensable in a correct diet. The consumption of free sugars was introduced in our diet relatively recently, and this was possible thanks to the industrial production of sugar, at first extracted from sugar cane and subsequently from sugar beets. Starting from the seventies of the last century, the introduction of HFCS, associated to the increasing use of SSBs, has led to a significant increase in the consumption of free sugars, including fructose. Young people are the heaviest consumers of free sugars and fructose. Fructose has specific metabolic pathways and, directly or through its metabolites, may promote the development of arterial hypertension and metabolic syndrome. No epidemiological or clinical studies are available in children that specifically demonstrate the effects of fructose. In fact, first of all, fructose is practically never consumed alone and secondly, it is rapidly removed from the circulation, so it cannot be dosed properly. On the other hand, there are numerous epidemiological studies, even in children, on the association between fructose and glucose consumption introduced by drinking SSBs and the presence of cardio-metabolic risk factors. The mixture of these two carbohydrates is particularly “explosive” as fructose and glucose, each acting through different mechanisms, can contribute synergistically to the development of cardiovascular disease. Vascular alterations at the base of these pathologies start in pediatric age and it would be therefore reasonable to start in time to prevent them. Given the large increase in the consumption of foods and beverages with added fructose in recent decades by children and adolescents, it is reasonable to think that increased fructose intake currently represents a real problem for the health of the younger generations, even if specific studies on this subject have not yet been published. A limitation of the consumption of free sugars and fructose should be among the first and most important measures to be taken for an effective prevention of increased cardiovascular risk.

## Author Contributions

SG and MG conceptualized and designed the review, drafted the initial manuscript, and reviewed and revised the manuscript. GL and AO prepared the tables and performed the reference search. GP reviewed and revised the manuscript. All authors have read and agreed to the published version of the manuscript.

## Conflict of Interest

The authors declare that the research was conducted in the absence of any commercial or financial relationships that could be construed as a potential conflict of interest.

## Publisher's Note

All claims expressed in this article are solely those of the authors and do not necessarily represent those of their affiliated organizations, or those of the publisher, the editors and the reviewers. Any product that may be evaluated in this article, or claim that may be made by its manufacturer, is not guaranteed or endorsed by the publisher.
